# Reaffirming Indigenous data sovereignty in New Mexico as a result of COVID-19

**DOI:** 10.3389/fpubh.2025.1302655

**Published:** 2025-06-03

**Authors:** William O. Carson, Felina M. Cordova-Marks, Lydia L. Jennings, Stephanie Russo Carroll

**Affiliations:** ^1^Homelands of the O’odham and Contemporary Lands of the Yaqui Peoples, Mel and Enid Zuckerman College of Public Health, University of Arizona, Tucson, AZ, United States; ^2^Homelands of the O’odham and Contemporary Lands of the Yaqui Peoples, Native Nations Institute, Udall Center for Studies in Public Policy, University of Arizona, Tucson, AZ, United States; ^3^Ohkay Owingeh; ^4^Hopi Tribe; ^5^Department of Environmental Studies, Dartmouth College, Hanover, NH, United States; ^6^Pascua Yaqui Tribe, Wixárika; ^7^Native Village of Kluti-Kaah

**Keywords:** New Mexico, Arizona, tribal governance, indigenous data sovereignty, COVID-19, data sharing, public policy

## Abstract

Despite New Mexico’s history of working with and enhancing collaboration with the 23 Tribes in the state, data sharing and collaboration with Tribes was poor during the COVID-19 pandemic. New Mexico’s policies of state collaboration with Tribes conflicts with the principles of Indigenous Data Sovereignty and fails to recognize Tribal public health authorities. New Mexico state agencies limited what data Tribes and Tribal Organizations received, resulting in the suppression of Tribes’ inherent rights. This policy brief concludes with recommendations for the state of New Mexico to respect Tribal sovereignty, uphold the tenants of Indigenous Data Sovereignty, restore trust with Tribes, and support increased capacity and capability of Tribes.

## Introduction

During the COVID-19 pandemic, American Indian populations saw rates of infection up to four times that of the general population (1). Decades of federal and state government underfunding for health care services were related to the higher infection rates faced by federally recognized Tribes (2). In New Mexico, the governor stated COVID-19 could cause Tribes to be “wiped out” (3). These claims, in part, created an environment where all Indigenous people within the state were to be viewed with suspicion. This hysteria contributed to a scandal around racial discrimination against American Indian people in New Mexico. We also observed massive challenges around data sharing and access to COVID-19 data for Tribes.

New Mexico’s history and contemporary experiences with Tribes appeared to go beyond funding and infrastructure issues. Twenty-three federally-recognized Tribes share a geography with New Mexico, and American Indian and Alaska Native people comprise 12.4% of the state population or approximately 263,615 per the 2020 US Census (4). Tribes in New Mexico include 19 Pueblos, 3 Apache Tribes, and the Navajo Nation. The state, given its location in the United States (US), has had a relationship of varying respect and cordiality with the Tribes in the state since its time as a territory. The state worked with Tribes throughout the pandemic to mitigate the pandemic. However, New Mexico dictated all the state level data around COVID-19, limiting involvement of Indigenous Public Health Authorities. As a result, this became a situation where the state was violating the principles of Indigenous Data Sovereignty (IDSov).

IDSov is a movement where Indigenous Peoples, including Tribes in the US, maintain rights over data about their people, land, cultures, and interests (5). Indigenous Peoples’ data, in this case, means any data that is created by Indigenous People or data that concerns Indigenous Peoples as collectives, their citizens and community members, and their lands (5). Tribes enact IDSov through Indigenous Data Governance (IDGov), an extension of the rights and practices that originate in the sovereignty of nation states (6). This commentary reviews ways the state of New Mexico struggled to uphold the rights of Tribes within its borders to access, use, and govern data during the pandemic and proposes ways to improve relationships going forward.

### Public health authorities

Tribes within the US have always been public health authorities, with that right and responsibility reaffirmed by the U.S. Code of Federal Regulations (7). Yet, only six Tribes have accredited public health departments, with zero in New Mexico (8). The lack of formal public health infrastructure can lead to situations where Tribal communities’ health services are overwhelmed in extreme circumstance, such as a pandemic, and limited resources may also limit an emergency response (9). Thus, the partnerships and relationships Tribes have with federal, state, and non-Tribal organizations are key (10). The federal and state governments show varying degrees of recognition of Tribal public health authority. These issues have led to situations where Tribes within the US are unable to access vital public health data despite the inherent rights of Indigenous Peoples. The United Nations Declaration on the Rights of Indigenous Peoples (UNDRIP) reaffirms the rights of Indigenous Peoples to self-determination, self-governance, and to maintain and strengthen their political, legal, social, and cultural institutions, which includes a right to public health data directly and indirectly involving them (11). Federally and state recognized Tribes within the US need access to data concerning their people and communities to exercise the right to self-determination and to address health inequities (12). Indigenous Peoples have the right to define the data to which they relate or link; possess, use, and control data concerning themselves; and govern access to data (12). These rights have not always been acknowledged by the US (13). Tribes are recognized as public health authorities through case law, Tribal codes, customary law and federal statute (13). Despite the inherent and recognized authority, gaps remain in Tribal public health infrastructure, with limited capacity across the country, as well as limited acknowledgement and recognition by states such as New Mexico.

In 2010, Congress amended the Indian Health Care Improvement Act (IHCIA) to permanently fund Tribal epidemiology centers (TECs) to collect and evaluate data, assist Tribes and Tribal organizations in health status objectives, and provide disease surveillance, among other tasks (14). The IHCIA also recognized TECs as public health authorities with access to federally available data, data sets, monitoring systems, delivery systems, and other protected health information (14). For example, the Albuquerque Area Southwest Tribal Epidemiology Center (AASTEC) serves 27 Tribes within the Indian Health Service Albuquerque Area which includes all 23 Tribes in New Mexico as well as those in Southern Colorado, Southern Utah, and West Texas (15). When state and federal agencies do acknowledge Tribes and TECs as public health authorities, they often stop short of defining the obligations that the agencies have when working with Tribal governments, Tribal health departments and TECs (10). As a result, AASTEC was left out of key COVID-19 collaborations between state and Tribes.

### Early instances of non-tribal organizations acquiring tribal specific data

Early on in the pandemic, it was found that COVID-19 affected Indigenous populations at greater rates compared to the general population (16–18), and while this is helpful for public health and medical professionals, it can also create unexpected tensions and consequences when in the hands of people who are less familiar with what these connections mean. The most infamous cases occurred at the largest private hospital system in the Albuquerque area, where findings from multiple researchers as well as in news reports were used to discriminate against Pueblo people. According to an initial report in ProPublica, the Department of Justice launched an investigation into Lovelace Women’s Hospital to determine if they had racially discriminated against Pueblo patients. The hospital implemented a policy that forcibly separated Pueblo mothers from their newborns for up to 3 days to ensure they were COVID-19 negative (19). The “Pueblo Lists,” only identifying areas with reservations and Pueblos, classified ZIP codes with fewer than seven cases as hot spots (19). The investigation argued that Lovelace specifically targeted Pueblo villages as hot spots for COVID-19 and determined this as a discriminatory practice against individual Indigenous people. In a follow up report by ProPublica, a second investigation was launched by the state to determine the extent of racial profiling at the hospital (20). This also concluded that the hospital violated patients’ rights by racially profiling Indigenous mothers and forcibly separating them from their children (21). One key question that has arisen from this is how were hospitals accessing readily available ZIP code level COVID-19 data, including on Tribal lands; thus, infringing on Tribal Sovereignty, within the state? What other ways were COVID-19 data for Tribes being used, counter to their needs, intention, and purposes?

In the academic world, researchers constructed a complex analysis using 372 ZIP codes within the state to argue that Indigenous populations were at greater risk for COVID-19 compared to the general population (22). News agencies such as New Mexico Political Report received COVID-19 cases by Tribal Nation affiliation through a simple data request (23). This organization reported on data concerning the Pueblo peoples in the state that included case breakdowns by Tribal Nation. These examples depict how New Mexico policies allowed non-Tribal entities to freely access Tribal data while at the same time, limiting data sharing and case reporting with Tribes, Tribal organizations, and the Albuquerque Area Southwest Tribal Epidemiology Center (AASTEC) (24, 25). Documentation of the collaboration efforts with Tribal Nations did not specify any data sharing agreements, nor if Tribal Nations had access to raw data for internal analysis or requesting assistance with the state or AASTEC. Notably, the state had agreements with Indian Health Service but none with AASTEC, a crucial public health authority which New Mexico Tribes work with on public health issues within their communities (25). This creates a potential conflict with the principles of IDSov and is further exacerbated by the state working in Pueblo lands without consent of the Pueblos.

Leadership at the Pueblo of Zia claimed that contact tracers with the state of New Mexico were notifying potentially infected or contacts of infected people on reservations without informing Tribal governments of their actions (26). Officials were first made aware of contact tracing efforts through Pueblo members rather than notification from state agencies (26). They claimed the state conducted contact tracing within Tribal borders without consulting first. This circumvention of government-to-government relationships went against prior policy work and against the principles of IDSov. These examples highlight challenges and limitations of current laws, policies, and practices in place for collaboration with Tribes.

### New Mexico’s state tribal collaboration act of 2009

New Mexico has policies to promote positive government-to-government relationships with Tribes. In 2009, the state passed the State Tribal Collaboration Act (STCA), that requires every state agency to implement policies of active collaboration and communication with Tribes and promote positive government-to-government relationships between agencies and Tribes (27). These documents can vary by department but reflected the state of the law as seen pre-Affordable Care Act (ACA). In the New Mexico Department of Health (New Mexico DOH) Tribal collaboration document, the intention of the new policy is to “build-upon previously agreed-upon processes when the Agency initiates programmatic actions that have Tribal implications” (28). These documents have not been updated since 2009 and as a result, do not reflect the status of public health infrastructure in Tribal communities today. COVID-19 further revealed gaps in the patchwork group of policies as the pandemic led to recurrent issues around data sharing and access, where non-Tribal organizations could access Tribal data while Tribes had little to no communication or access.

### COVID-19 data sharing with tribes

Like all other state and federal agencies, New Mexico maintains detailed plans for how they set up their Emergency Operations Center (EOC) in times of a public health emergency, most recently updated in 2014 (29). The emergency operations plan (EOP) developed by the state is sixty-two pages long and has just three paragraphs dedicated to coordination with Tribes. The EOP fails to detail what Tribal governments and Tribal organizations such as AASTEC are entitled to during a request for information (28).

The state plans for COVID-19 response did not mention data sharing with Tribes and AASTEC, despite their designation as public health authorities empowered to collect data, develop policies and programs, and conduct surveillance within their Tribal bounds or service areas (7, 14). Tribes are mentioned just twice in the 24-page addendum specific to the COVID-19 Pandemic (30). As a result, the state minimized communication with Tribes and excluded AASTEC. The state Indian Affairs Department also maintains a COVID-19 Tribal response plan failed to mention data sharing, only ways Tribes can be notified, as well as prevention and mitigation measures (31). In follow up reports by the New Mexico DOH Tribal Liaison, details on the distribution of case lists to Tribes are listed, but there is no mention of agreements for data access, sharing, or privacy protections with Tribes (24, 25, 32). In a discussion with a local news agency, a former government official at the Pueblo of Acoma stated that some Tribes do not have the necessary personnel to properly interpret the data the state would theoretically be sending them even if the data was accessible, which it currently is not (9).

New Mexico state agencies collaborate with Tribes in their COVID response at their own discretion. The New Mexico DOH policy is written to encourage consultation or collaboration through informal channels (28). While the state of New Mexico annual reports discuss government-to-government relationships with Tribes, they do not mention specific collaboration or data sharing agreements between the state and Tribe(s) (24, 25, 33). As of the July 2024 report, there have been no further updates to the New Mexico COVID-19 response that specify data sharing agreements with Tribes for COVID-19 (25). Despite AASTEC’s status as a Tribal Public Health Authority, neither AASTEC nor a general mention of Tribal Public Health Authorities are mentioned in New Mexico policy documents (27–29). Tribes may not have the capacity to analyze and interpret public health data but that is one purpose of TECs. If Tribal Nations choose to engage, AASTEC can help act to fulfill epidemiology related tasks for Tribes, so they are not so dependent on the state. This is made more necessary by how NM overshared data about Tribal Nations with non-Tribal organizations while also limiting Tribal Nations’ COVID-19 data access, thus reducing their decision-making ability of Tribal emergency operations command.

Collaboration between states and Tribes is difficult in times of general operating procedure, more so during emergencies. Thus, preparation in terms of policy and infrastructure during general operating times is key. As seen with COVID-19 data sharing, laws and policy in Arizona allowed for greater recognition and implementation of Indigenous Data Governance compared to that of New Mexico.

### COVID-19 tribal data policy distinctions between New Mexico and Arizona

The state of Arizona is home to 22 Tribes and more than 386,000 American Indian and Alaska Native people (34). The politics of Arizona are often put into contrast with the state of New Mexico, with more progressive policies coming out of Santa Fe as opposed to Phoenix due to having democratic majorities in the state house compared to a split government in Arizona. There are no shortages of disputes between Arizona and the Tribes in the state, with the most recent example being where Arizona was victorious in the Supreme Court in a case limiting water rights of the Navajo Nation. However, the goal of this section is to highlight that IDSov is not a left vs. right issue but instead a different spectrum of pro- or anti-Tribal sovereignty.

Arizona, like New Mexico, has a consultation agreement between state agencies and Tribes with which it shares a geography. The Arizona Department of Health’s consultation policy articulates communication recommendations, states respect for Tribal sovereignty, and has defined time frames for response to Tribes’ requests (35). Arizona’s Department of Emergency and Military Affairs’ (AZDHSEM) documents define 15 policies and procedures that the agency will follow that empower Tribal governments and respect Tribal sovereignty (36).

**Figure 1:**
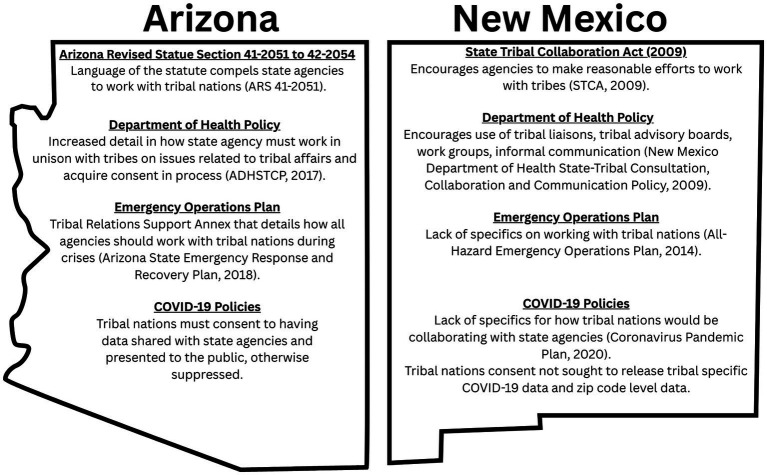
Comparison of Arizona vs. New Mexico state tribal collaboration policies.

Arizona’s EOP, in direct comparison to New Mexico, contains extensive information for the EOC on how they are to collaborate with Tribes (37). A significant difference observed is that publicly available COVID-19 data was more readily accessible in New Mexico compared to Arizona. For published cases of COVID-19, the state of Arizona suppressed data from ZIP codes that overlay Tribes (38). This is shown through marking ZIP codes with the code “TRIBAL” on the map (38). New Mexico made no such distinction when COVID-19 data was being actively tracked. Figure 1 is a brief comparison of the statutes and policies in the states of New Mexico and Arizona.

### Policy improvements to affirm tribal sovereignty

One of the main takeaways from the COVID-19 pandemic, when viewed through the lens of IDSov, was that Indigenous Peoples were seen as victims due to poor health, poor infrastructure, and other such examples. This deficit gaze of Tribal Nations has been reported on, including a comparison of how the New York Times reported on the policies of Navajo Nation, compared to reporting by the Navajo Times, a newspaper on the reservation (39). Western institutions painted stories more focused on what was missing and the pitying of Indigenous Peoples while Indigenous sources often highlighted the potential for improvement and resilience. This deficit perspective also appeared in the New Mexico government response, newspaper reports, and academic research. Tribal Nations and AASTEC had limited access to raw data, which is vital for informed decision making, and lacked collaborative relationships with the state. Indeed, international organizations have called for open data access and updated data sharing agreements to ensure that scientific discovery is as quick as possible and that governments are able to govern (40). The current STCA guidelines need to be revised to respect the principles of IDSov and collaboration. We suggest the following revisions.

#### Revise the New Mexico emergency operations plans to include specifics for tribe consultation and collaboration

The state’s response strategy to COVID-19 should have been done collaboratively with Tribes as the language in the Department of Homeland Security and Emergency Management STCA Communication and Collaboration Policy clearly lays out in Section V, A. 5. B that Tribal consultation should occur before action that impacts Tribal governments and citizens (37). EOP guidelines should be targeted and specific in the obligations of the state to Tribes. Tribe.

#### Compel collaboration by state agencies in STCA policies

The New Mexico STCA includes informal communication in the same list as work groups, advisory boards, and liaisons. Section V., Part A, line number 4 should be changed to better match article 19 on collaboration, and article 24, section 2 on the right to health reaffirmed in the UN Declaration on the Rights of Indigenous Peoples (11).

Language should be changed so informal communication is never a replacement for formal requests or formal consultation. In addition, this new language empowers Tribal health organizations like AASTEC.

#### Expand the STCA to incorporate the tribal public health authorities

In the STCA, New Mexico does not define the role that AASTEC has in consultation and data sharing (28). This may be because the STCA was passed in 2009 and the IHCIA was not passed until 2010. In Arizona it is stated that: “ADHS may also provide written notice and a solicitation for feedback to non-Tribal or other American Indian organizations such as the Arizona Advisory Council on Indian Health Care, the Inter-Tribal Council of Arizona, Inc., the Indian Health Service Area Offices in Arizona and Urban Indian Health programs, and other state agencies. Such communications do not substitute for direct consultation with the Tribes in Arizona” (35). New Mexico must acknowledge AASTEC as a public health authority. While they are not a Tribe, AASTEC is a federally authorized public health authority and one which has relationships with every Tribe within the state of New Mexico. Including AASTEC in future policy updates in ways that align with IDSov and Tribal sovereignty will both respect their authority and allow for Tribes to have more resources at their disposal to build capacity and react to public health emergencies in a timely manner, while also ensuring communication between state and Tribes is collaborative instead of dictation.

#### Strengthen partnerships between tribal epidemiology centers and tribes

Finally, while we have mentioned that the state of New Mexico must recognize the public health authority of AASTEC, this alone will not resolve any issues. TECs are public health authorities staffed by public health professionals but TECs are not Tribes and therefore must respect IDSov principles when working with any Tribal Data (5). AASTEC, like all TECs, must develop agreements with all Tribes within their service area to work with Tribal health data. Written agreements such as this may aid in growing public health infrastructure for Tribes and develop state level recognition for AASTEC.

## Conclusion

The COVID-19 response in New Mexico shows a fundamental failing with the current state-Tribal collaboration policies. In the first year of the pandemic, Tribes had no governance authority of their data and were limited in what they received. In the following years, New Mexico remained without data sharing agreements for Tribes and AASTEC despite further collaboration with a limited numbers of organizations (24, 25). As a result, the data failed to be used in a way that benefits the Tribes, and arguably the state, of New Mexico.

The STCA should cement the obligation that state agencies must consult and collaborate with Tribes. Currently, guidance reinforces a paternalistic relationship between state and Tribes by refusing to acknowledge the sovereignty of Tribes and federally funded support services. The lack of clarity has a potential impact on how all agencies interact with Tribes daily and in times of crisis. COVID-19 is only one public health emergency, we know there will always be more to come. To prevent these issues in the future, New Mexico should rewrite the STCA in a joint workgroup with the Tribes and Tribal organizations in the state. Furthermore, New Mexico should publish new guidance that explicitly defines data sharing protocols that include American Indian and Alaska Native data. Respect for Tribal sovereignty should be promoted by the state through co-development of policy with Tribes and Tribal organizations so that future crises can be addressed more effectively.

## References

[1] Rodriguez-Lonebear D Barceló NE Akee R Carroll SR. American Indian reservations and Covid-19: correlates of early infection rates in the pandemic. J Public Health Manag Pract. (2020) 26:371–7. doi: 10.1097/Phh.0000000000001206, PMID: 32433389 PMC7249493

[2] Carroll SR Akee R Chung P Cormack D Kukutai T Lovett R. Indigenous Peoples' data during Covid-19: from external to internal. Front Sociol. (2021) 6:617895. doi: 10.3389/Fsoc.2021.617895, PMID: 33869569 PMC8022638

[3] abc News. Katherine Faulders or. New Mexico's Governor Warns Tribal Nations could be 'Wiped Out' By Coronavirus. Available online at: https://abcnews.go.com/politics/mexicos-governor-warns-tribal-nations-wiped-coronavirus/story?id=69884997

[4] Staff Ac. New Mexico Population Grew 2.8% Last Decade. Accessed September 9, 2023. Available online at: https://www.census.gov/library/stories/state-by-state/new-mexico-population-change-between-census-decade.html

[5] Carroll SR Herczog E Hudson M Russell K Stall S. Operationalizing the care and fair principles for indigenous data futures. Sci Data. (2021) 8, 1–6. doi: 10.1038/s41597-021-00892-0, PMID: 33863927 PMC8052430

[6] Carroll SR Rodriguez-Lonebear D Martinez A. Indigenous data governance: strategies from United States native nations. Data Sci J. (2019) 18, 1–15. doi: 10.5334/Dsj-2019-031, PMID: 34764990 PMC8580324

[7] Indian health care improvement act, title 25 U.S.C. § 1621m (2010). Indian Health Service, Department of Health and Human Services.

[8] Increase the Number of Tribal Public Health Agencies that are Accredited — Phi-03. U.S. Department Of Health And Human Services. Avaialble online at: https://health.gov/healthypeople/objectives-and-data/browse-objectives/public-health-infrastructure/increase-number-tribal-public-health-agencies-are-accredited-phi-03

[9] Sims J. Tribal data, power and information in the COVID era. The Paper. Available online at: https://abq.news/2021/05/tribal-data-power-and-information-in-the-covid-era/

[10] O’connell MC Abourezk C. Facilitating the urgent public health need to improve data sharing with tribal epidemiology centers. Public Health Rep 138:00333549231152197. doi: 10.1177/00333549231152197 PMC10515977 36734206

[11] Assembly UG. United Nations declaration on the rights of indigenous peoples. Un Wash. (2007) 12:1–18.

[12] Hudson M Carroll SR Anderson J Blackwater D Cordova-Marks FM Cummins J. Indigenous Peoples' rights in data: a contribution toward indigenous research sovereignty. Perspect Front Res Metrics Anal. (2023) 8:1173805. doi: 10.3389/Frma.2023.1173805 PMC10192690 37215248

[13] Hoss A. A framework for tribal public health law. Nev Lj. (2019) 20:113. doi: 10.2139/ssrn.3428572

[14] Hoss A Ransom MM Penn MS. Tribal epidemiology centers designated as public health authorities under the health insurance portability and accountability act. (2015). Centers for Disease Control and Prevention.

[15] Aastec. Who We Serve. Accessed September 11, 2023. Availabe online at: https://www.aastec.net/who-we-serve/

[16] Jackson NR Zeigler K Torrez M Makino Y Adolphi NL Lathrop S. New Mexico’s Covid-19 experience. Am J Forensic Med Pathol. (2021) 42:1–8. doi: 10.1097/Paf.0000000000000664, PMID: 33416234 PMC7870043

[17] Hicks JT Burnett E Matanock A Khalil G English K Doman B. Hospitalizations for Covid-19 among American Indian and Alaska native adults (≥ 18 years old)—New Mexico, march–September 2020. J Racial Ethn Health Disparities. (2023) 10:56–63. doi: 10.1007/s40615-021-01196-0, PMID: 35060084 PMC8776374

[18] Kakol M Upson D Sood A. Susceptibility of southwestern American Indian tribes to coronavirus disease 2019 (Covid-19). J Rural Health. (2020) 37:197–9. doi: 10.1111/jrh.12451, PMID: 32304251 PMC7264672

[19] Furlow B. A Hospital’s Secret Coronavirus Policy Separated Native American Mothers from their Newborns. Albuquerque: Propublica Available online at: https://www.propublica.org/article/a-hospitals-secret-coronaviruspolicy-separated-native-american-mothers-from-their-newborns (Accessed March 16, 2021). (2020).

[20] Furlow B. State investigating hospital with coronavirus policy that profiled pregnant native American mothers and separated them from newborns. New Mexico: Propublica And In Depth. Available online at: https://www.propublica.org/article/state-investigating-hospital-with-coronavirus-policy-that-profiled-pregnant-native-american-mothers-and-separated-them-from-newborns

[21] Furlow B. Federal Investigation Finds Hospital Violated Patients’ right by profiling, separating native mothers and newborns. New Mexico: Propublica and in Depth (2020).

[22] Huyser KR Yang T-C Horse AJY. Indigenous peoples, concentrated disadvantage, and income inequality in New Mexico a zip code-level investigation of spatially varying associations between socioeconomic disadvantages and confirmed Covid-19 cases. J Epidemiol Community Health. (1979) 75:1044–9. doi: 10.1136/jech-2020-215055, PMID: 33757989

[23] Childress M. Covid-19 has spread to Most New Mexico tribes. The New Mexico Political Report. Available online at: https://nmpoliticalreport.com/2020/05/14/covid-19-has-spread-to-most-new-mexico-tribes/1

[24] State-tribal collaboration act July 31, 2022 agency report (2022). New Mexico Department of Health.

[25] State-tribal collaboration act July 31, 2023 agency report (2023). New Mexico Department of Health.

[26] Bichelle RE. Pandemic complicates tribes’ quest for data sovereignty. Kunc. Available online at: http://www.kunc.org/health/2020-07-13/pandemic-complicates-tribes-quest-for-data-sovereignty

[27] The State Tribal Collaboration Act, Sb 196, § 3 (2009). State of New Mexico.

[28] New Mexico Department of Health State-Tribal Consultation, Collaboration and Communication Policy—§ V. (2009). New Mexico Department of Health.

[29] New Mexico Department of Health All-Hazard Emergency Operations Plan—§ Coordination with Tribal Entities (2014). New Mexico Department of Health.

[30] New Mexico Department of Health All-Hazard Emergency Operations Plan Hazard Annex F: Outbreaks Coronavirus Pandemic Plan—§ Purpose, Scope, Situation and Assumptions (2020). New Mexico Department of Health.

[31] Tribal Response Plan Covid-19 State of New Mexico (2020). New Mexico Indian Affairs Department.

[32] 2021 Tribal liaison report, quarter 1, § announcements, Accomplishments, & Other Updates (2021). New Mexico Indian Affairs Department.

[33] State-Tribal Collaboration Act July 31, 2021 Agency Report (2021). New Mexico Department of Health.

[34] Leading Causes Of Death And Health Disparities Among American Indian/Alaska Natives (2021). Arizona Department of Health Services.

[35] Arizona Department Of Health Services Tribal Consultation Policy (2017). Arizona Department of Health Services.

[36] McGuire M. Tribal Consultation Policy. Arizona Department of Emergency and Military Affairs: State of Arizona. (2018). Retrieved from https://dema.az.gov/resources/dema-tribal-consultation

[37] Arizona State Emergency Response and Recovery Plan—§ Tribal Relations Support Annex (2018). Arizona Department of Emergency and Military Affairs.

[38] COVID-19 Data; Cases by zip code (2022). Arizona Department of Health Services.

[39] Camarillo E Kunze S Pollard C. How the media framed the Covid-19 crisis on native nations: a case comparison of *the New York Times* and the *Navajo times*. Soc Sci Q. (2024) 105:54–67. doi: 10.1111/Ssqu.13331

[40] Pickering B Biro T Austin CC Bernier A Bezuidenhout L Casorrán C. Radical collaboration during a Global Health emergency: development of the Rda Covid-19 data sharing recommendations and guidelines. Open research. Europe. (2021) 1:1. doi: 10.12688/openreseurope.13369.1, PMID: 37645170 PMC10446077

